# Tetraspanin CO-029 Inhibits Colorectal Cancer Cell Movement by Deregulating Cell-Matrix and Cell-Cell Adhesions

**DOI:** 10.1371/journal.pone.0038464

**Published:** 2012-06-05

**Authors:** Qiusha Guo, Bing Xia, Feng Zhang, Mekel M. Richardson, Minghao Li, Julian S. Zhang, Feng Chen, Xin A. Zhang

**Affiliations:** 1 Vascular Biology and Cancer Centers and Departments of Medicine and Molecular Science, University of Tennessee Health Science Center, Memphis, Tennessee, United States of America; 2 Department of Gastroenterology, Zhongnan Hospital, Wuhan University Medical School, Wuhan, China; 3 Internal Medicine, Renal Division, Washington University School of Medicine, St. Louis, Missouri, United States of America; National Taiwan University Hospital, Taiwan

## Abstract

Alterations in tetraspanin CO-029 expression are associated with the progression and metastasis of cancers in the digestive system. However, how CO-029 promotes cancer metastasis is still poorly understood. To determine the mechanism, we silenced CO-029 expression in HT29 colon cancer cells and found that the CO-029 knockdown significantly reduced cell migratory ability. The diminished cell migration was accompanied by the upregulation of both integrin-dependent cell-matrix adhesion on laminin and calcium-dependent cell-cell adhesion. The cell surface levels of laminin-binding integrin α3β1 and fibronectin-integrin α5β1 were increased while the level of CD44 was decreased upon CO-029 silencing. These changes contribute to the altered cell-matrix adhesion. The deregulated cell-cell adhesion results, at least partially, from increased activity of cadherins and reduced level of MelCAM. In conclusion, CO-029 functions as a regulator of both cell-matrix and cell-cell adhesion. During colon cancer progression, CO-029 promotes cancer cell movement by deregulating cell adhesions.

## Introduction

Colorectal cancer, one of the most common cancers, has high mortality [1]. Patients with metastasis to distant organs such as liver and lungs suffer extremely poor prognosis. Therefore, understanding the cellular and molecular mechanisms of colorectal cancer progression is critical for developing new strategies to improve the prognosis and survival rates for colorectal cancer patients.

Tetraspanins regulate a variety of physiological and pathological processes, and some tetraspanins are associated with cancer progression and metastasis [2]–[11]. Human tetraspanin CO-029 and its rat homologue, D6.1A, were initially reported as a tumor-associated antigen expressed in gastric, colorectal, and pancreatic cancer cells and exert tumor progression-promoting activity [12]. CO-029 expression is frequently upregulated in hepatocellular carcinoma [13]. The expression level of D6.1A is markedly increased, relative to the one in a differentiated parental line, in a dedifferentiated rat hepatoma cell line [14]. The simultaneous expression of integrin α6β4 and D6.1A in nonmetastasizing rat pancreatic adenocarcinoma cell line BSp73AS facilitates the liver metastasis of this line [15]. CO-029 also exhibits a higher expression level in metastatic colon carcinoma cells, compared with the level in primary colon cancer cells [16]. A possible mechanism for the prometastatic activity of CO-029 is its association with integrins or tetraspanins, both of which affect cell motility. D6.1A is associated with integrins α3β1, α6β1, and α6β4 after protein kinase C (PKC) activation [15], [17]. In metastatic pancreatic and colorectal carcinoma cell lines, the activation of PKC enhances the colocalization of CO-029 and tetraspanin CD151 with integrin α6β4 in tumor cells, promotes the internalization of this integrin-tetraspanin complex, decreases cell-matrix adhesion on laminin 332, and increases cell migration [18]. Furthermore, D6.1A-overexpressing tumor cells release the exosomes that contain D6.1A, and these exosomes induce angiogenesis to facilitate tumor dissemination [19]. A recent study showed that E-cadherin and p120-catenin antagonize CO-029 promoted migration of Isreco colon cancer cells [20]. These studies strongly suggest an important role for CO-029 in the progression and metastasis of tumors in the digestive system. To determine the mechanism by which CO-029 promotes tumor progression and metastasis, we silenced the expression of CO-029 in HT29 human colon adenocarcinoma cells. By combining in vitro and in vivo experiments, we found that the loss of CO-029 significantly attenuated cell motility and altered the balance of cell-cell and cell-matrix adhesions, leading to the decreased metastatic potential of tumor cells.

## Materials and Methods

### Cell Culture, Antibodies, Extracellular Matrix Proteins, and Other Reagents

HT29 human colorectal adenocarcinoma cell line was obtained from ATCC (Manassas, VA) and cultured in DMEM supplemented with 10% fetal bovine serum, 100 units/ml penicillin, and 100 µg/ml streptomycin.

The antibodies used in this study were intergrin α1 mAb TS2/7, intergrin α2 mAb IIE10, intergrin α3 mAb A3X8, intergrin α5 mAb BIIG2, intergin α6 mAb A6BB, intergrin β1 mAb TS2/16, intergrin β4 mAb 439-9B (BD Pharmingen, San Diego, CA), CD9 mAbs C9BB [21] and Mab7, CD63 mAb 6H1 [22], CD81 mAb M38, CD82 mAb M104, CD151 mAbs 5C11 and TS151r [23], CO29 mAb NS1116 (kindly provided by Dr. Dorothee Herlyn of the Wistar Institute), E-cadherin mAb (Santa Cruz Biotechnology, Santa Cruz, CA), EpCam mAb VU1D9 (Cell Signaling, Danvers, MA), EWI2 mAb 5E8 (kindly provided by Dr. T. Schweighoffer of the Novartis Institute for Biomedical Research), and MelCam mAb P1H12 (Santa Cruz Biotechnology, Santa Cruz, CA). A mouse IgG2b was used as a negative control antibody (Sigma, Saint Louis, MO). The second antibodies used in this study were horseradish peroxidase-conjugated goat-anti-mouse IgG antibody (Sigma, Saint Louis, MO) and fluorescein isothiocyanate-conjugated goat-anti-mouse or -rat IgG antibody (Biosource International, Camarillo, CA).

The extracellular matrix proteins were reconstituted mouse basement membrane Matrigel (BD Biosciences, Mountain View, CA), mouse laminin 111 (Invitrogen, San Diego, CA), and human plasma fibronectin (Invitrogen, San Diego, CA). Other reagents were from Sigma if the source is not specified.

### RNA Interference and Retrovirus Preparation and Transduction

The CO-029 small hairpin RNA (shRNA) (TGCTGTTGACAGTGAGCGATGAATGAAACTCTCTATGAAATAGTGAAGCCACAGATGTATTTCATAGAGAGTTTCATTCACTGCCTACTGCCTCGGA), which targets the TGAATGAAACTCTCTATGAA sequence of human CO-029 mRNA, and control shRNA (RHS1703) were obtained from OpenBiosystems (Huntsville, AL). Another CO-029 shRNA that targets a different part of CO-029 mRNA sequence was obtained from OpenBiosystems (Materials & Methods S1). The vesicular stomatitis virus (VSV)-G protein expression vector pVSV-G (kindly provide by Dr. C. Stipp, University of Iowa) and shRNA were transduced into the GP293 packaging cells by Lipofectamine 2000 (Invitrogen, San Diego, CA). After 48 h, the virus particle-containing culture supernatant was harvested, and the cell debris was removed by passing the supernatant through a 0.45-µmol/L syringe filter. The purified viral stock was incubated with HT29 cells for transduction, and the HT29 cells transduced with control or CO-029 shRNA were selected with puromycin. The puromycin-resistant cells in CO-029 shRNA transductant were sorted by flow cytometry to enrich the CO-029-silenced cell population.

### Flow Cytometry

Cells were detached with 0.5% trypsin-EDTA, washed with phosphate-buffered saline (PBS) twice, blocked with 5% goat serum in DMEM at 4°C for 1 h, incubated with primary mAb, and then stained with FITC-conjugated goat-anti-mouse IgG at 4°C for 1 h. Stained cells were analyzed on a FACScan flow cytometer (BD Biosciences).

### Immunofluorescence

Cells were fixed with 3% paraformaldehyde at room temperature (RT) for 15 min, permeabilized with 0.1% Brij 98 at RT for 2–5 min, blocked with 20% goat serum at 4°C for 1 h, and incubated with primary mAbs at 4°C for 1 h, followed by staining with a secondary antibody at 4°C for 1 h. After each antibody incubation, the cells were washed three times with PBS. For immunofluorescent analysis, the cells were examined with an Axiophot fluorescent microscope (Carl Zeiss, Thornwood, NY), and images were captured using an Optronics digital camera.

### Immunoprecipitation and Western Blot

Immunoprecipitations were carried out as described previously [24]. Cells were lysed with 1% Nonidet P-40 lysis buffer at 4°C for 1 h. In some experiments, the cell surface was labeled with 0.5 mg/ml EZlink sulfo-NHS-LC biotin (Pierce) before cell lysis. After removing the insoluble material by centrifugation at 14,000× g and two runs of pre-clearance, lysates were incubated with primary mAb-absorbed protein A- and G-Sepharose beads (Amersham Biosciences, Piscataway, NJ) from 3 h to overnight at 4°C. The immunoprecipitates were washed with lysis buffer three times, dissolved in Laemmli sample buffer, heated at 95°C for 5 min, separated by SDS-PAGE, and then electrically transferred to nitrocellulose membranes (Bio-Rad, Hercules, CA). The membranes were sequentially blotted with primary Ab and horseradish peroxidase-conjugated second Ab (Sigma) for immunoblot experiments or horseradish peroxidase-conjugated extravidin (Sigma) for biotinylation experiments, followed by chemiluminescence (PerkinElmer Life Sciences, Waltham, MA).

### Cell Adhesion Assays

Cell-matrix adhesion assay was performed as described in our earlier study [25]. Briefly, 96-well microculture plates (BD Bioscience) were coated with mouse laminin 111 or fibronectin at 4°C overnight and then blocked with 1% heat-inactivated bovine serum albumin (Sigma) at 37°C for 1 h. Laminin 332 was prepared by culturing RAC-11P cells at confluence in the 96-well plates for 3 days as described earlier [26]. Cells were trypsinized and suspended in serum-free DMEM medium at a density of 1×10^4^ cells/ml, and 0.1 ml of the cell suspension was then added to each well. After incubation for 1 h at 37°C, unattached cells were removed by rinsing four times with PBS. The attached cells were visually counted.

Cell-cell adhesion was examined by using the hanging-drop aggregation assay as described earlier [27]. Cells were detached with 0.5% tripsin-EDTA and washed with PBS twice, then rendered into single-cell suspension by three gentle passes through a 27-gauge needle. The single-cell suspension of 1×10^4^ cells in 30 µl of solution was suspended as a hanging drop from the lid of a 24-well culture dish and allowed to aggregate overnight at 37^o^C in 5% CO_2_ with humidity. Complete DMEM was used for measuring total cell-cell adhesiveness, while calcium-free DMEM was for calcium-independent cell-cell adhesiveness. To assay the resistance of cell-cell adhesion to mechanical stress, the cells were subjected to shear force by passing them through a 200-µl pipet tip 10 times. Calcium-independent cell-cell adhesion was also measured under relatively mild mechanical stress [28]. Briefly, after washing with Puck's saline (5 mM KCl, 140 mM NaCl, 8 mM NaHCO3, pH 7.4), the single-cell suspension (1×10^5^ cells/ml) was incubated in 5% CO_2_ at 37°C overnight with agitation at 80 rpm on an orbital shaker. Cells were photographed either before or after mechanical stress. Cell-cell adhesiveness after shear stress was quantified as i) the percentage of aggregated cells and/or ii) the area covered by aggregates. Based on the count of single cells, the percentage of aggregated cells was calculated by using the formula % aggregation  =  [1– (number of single cells/number of total cells)] ×100. The surface area covered by the aggregates was measured by using ImageJ software to portray the degree of cell aggregation. Cell aggregate is defined as a cell clump containing four or more cells.

### Cell Migration Assays

Wound healing assay was performed as described in our earlier study [29]. Briefly, cells were seeded into individual wells of a 24-well culture plate. When the cells reached confluence, a cell monolayer was first treated with 10 µg/ml mitomycin C for 30 min to block mitosis and thus allow analysis of cell migration in the absence of cell proliferation. Then the cell monolayer was wounded with a sterile, 200- µl pipette tip. The medium and cell debris were removed and replenished by 2 ml of fresh medium. Cells were photographed by phase-contrast microscopy every 24 h after wounding. To evaluate “wound closure”, five areas along the wound were randomly chosen for calculating the average width for each well. Photos were taken at different time points by an Olympus inverted-phase contrast microscope.

Transwell cell migration assay was performed as described in our earlier study [30]. Briefly, the 8-µm pore size, 24-well Transwell inserts (BD Bioscience, Bedford, MA) were precoated with 10 µg/ml of laminin 111 or fibronectin onto the underside of the inserts at 4°C overnight and blocked with 0.1% heat-inactivated bovine serum albumin (BSA) at 37°C for 1 h. A total of 2×10^4^ cells in serum-free DMEM containing 0.1% heat-inactivated BSA in a volume of 300 µl was added to the upper chamber, and 500 µl DMEM containing 1% FBS were added to the lower chamber. The cells in transwells were incubated for 24 h at 37°C. The cells remaining on the upper surface of the inserts were removed by wiping with a cotton swab, and the cells that transmigrated through the pores were fixed and stained with Diff-Quik Stain Kit (Dade Behring, Inc., Newark, DE). Migration was quantified by counting the cells on the lower surface of the insert.

### Statistical Analyses

All experiments were performed at least four times. Data are presented as mean±SD. Statistical analysis was performed by Student’s *t*-test, and *P*<.05 was considered statistically significant.

## Results

### The Silence of CO-029 Expression in HT29 Colon Cancer Cells

**Figure 1:**
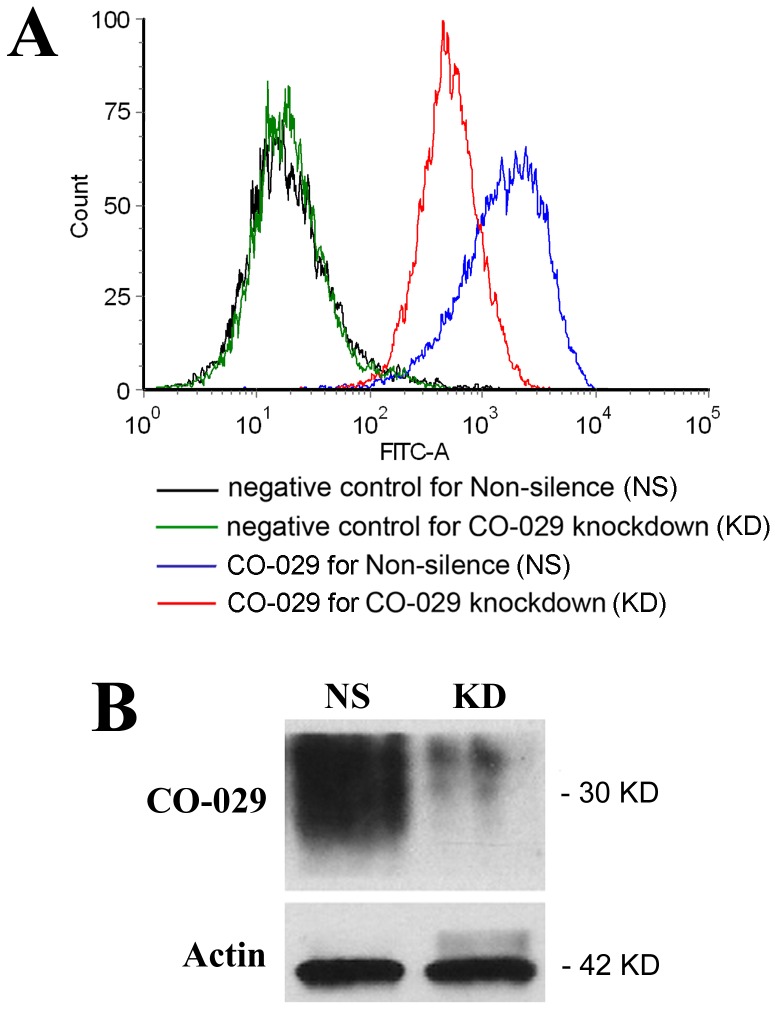
Silencing CO-029 expression. The CO-029 shRNA and nonsilencing shRNA were stably expressed in HT29 cells. (**A**) Flow cytometric analysis of CO-029 expression at the surface of HT29 cells that were transfected with nonsilencing (NS) or CO-029 shRNA (KD) constructs. The negative control mAb was murine IgG, and CO-029 mAb was NS1116. Mean fluorescent intensity (MFI) of CO-029 on KD cells was 62% less compared to that on NS cells. (**B**) Western blot analysis of CO-029 proteins in NS and KD HT29 cells. KD cells displayed a 90% of reduction in CO-029 proteins. β-actin: loading control.

To determine the mechanistic roles of tetraspanin CO-029 in tumor progression, we established a stable transductant in HT29 human colon adenocarcinoma cells [31], in which CO-029 expression was knocked down by shRNA. The silencing effects on the total cellular and cell surface CO-029 protein expression were assessed by using Western blot and flow cytometry, respectively (Figure 1). Compared with the transductant expressing control or nonsilencing (NS) shRNA, the transductant expressing CO-029 shRNA (KD) exhibited a substantial reduction of CO-029 expression at the cell surface (Figure 1A), ranging from approximately 60% to 90%, depending on the cell confluence status in each individual experiment, and a large loss of total cellular CO-029 proteins (Figure 1B), typically approximately 90%. The silence resulting from CO-029 shRNA was specific to CO-029 because the expression of many other surface proteins, especially other members of the tetraspanin superfamily, was not reduced (see following).

### The Silence of CO-029 Expression Impaired Migration of HT29 Cells

**Figure 2:**
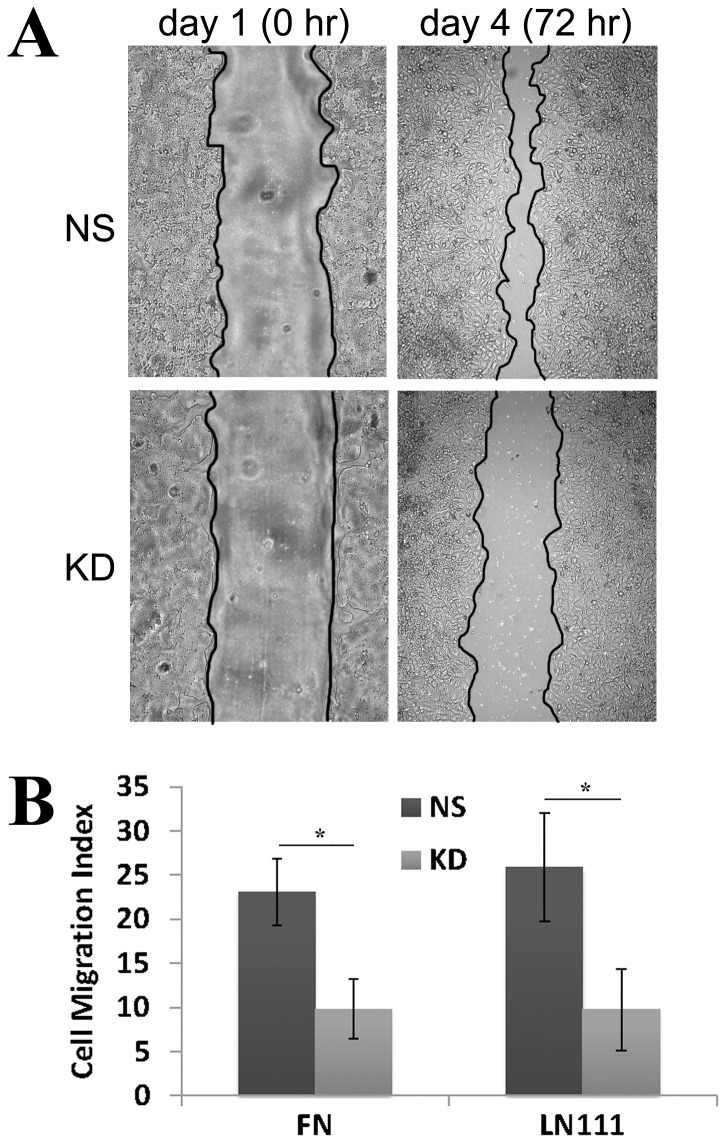
CO-029 silencing impaired colorectal cancer cell migration. (**A**) Wound healing assay. Compared with NS cells, wound closure was significantly impaired in the KD cells at 72 h after creation of wounds in confluent cell monolayers. (**B**) Transwell migration assay. The KD cells displayed impaired motility in transwell migration experiments. Data are displayed as mean±SD, n = 4. *P<.05.

We first assessed the effect of CO-029 silencing on cancer cell migration. To compare the cell migratory ability of NS and KD transductants, we performed 1) wound healing assay to analyze collective cell migration, i.e., cell-cell adhesion-dependent cell migration, and 2) transwell migration assay to analyze solitary cell migration, i.e., cell-cell adhesion-independent cell migration. In the wound healing experiments, the repopulation rate or healing process of the wounded monolayer was markedly reduced in CO-029 KD transductant cells, and such reduction lasted several days (Figure 2A and Figure S1A). The reduced wound healing was not resulted from slower cell proliferation of the KD cells because the experiments were performed in the presence of mitomycin. The impaired ability of KD cells in cell migration could also be observed in the transwell migration assay. Cell migration through the pored membrane filter onto extracellular matrix proteins such as laminin 111 and fibronectin was significantly decreased upon the silencing of CO-029 expression (Figure 2B and Figure S1B). These results indicate that the silence of CO-029 attenuates the general ability of cell migration and that the expression of CO-029 is required for strong or robust cell migration.

In addition to cell migration on 2-dimensional matrix, cell invasion is an important parameter to measure cell motility through 3-dimensional (3D) microenvironment for invasive and metastatic cancer cells. Cellular invasiveness was measured by the ability of HT29 transductant cells to invade through Matrigel, a 3D matrix similar to basement membrane, or type I collagen gel, a 3D matrix similar to connective tissue. We found no significant invasion of either group of HT29-KD cells through either of these 3D matrix environments (data not shown), suggesting that HT29 cells are not invasive, at least in this invasion assay and under the *in vitro* test condition.

### CO-029 Regulated Cell-matrix and Cell-cell Adhesion

**Figure 3:**
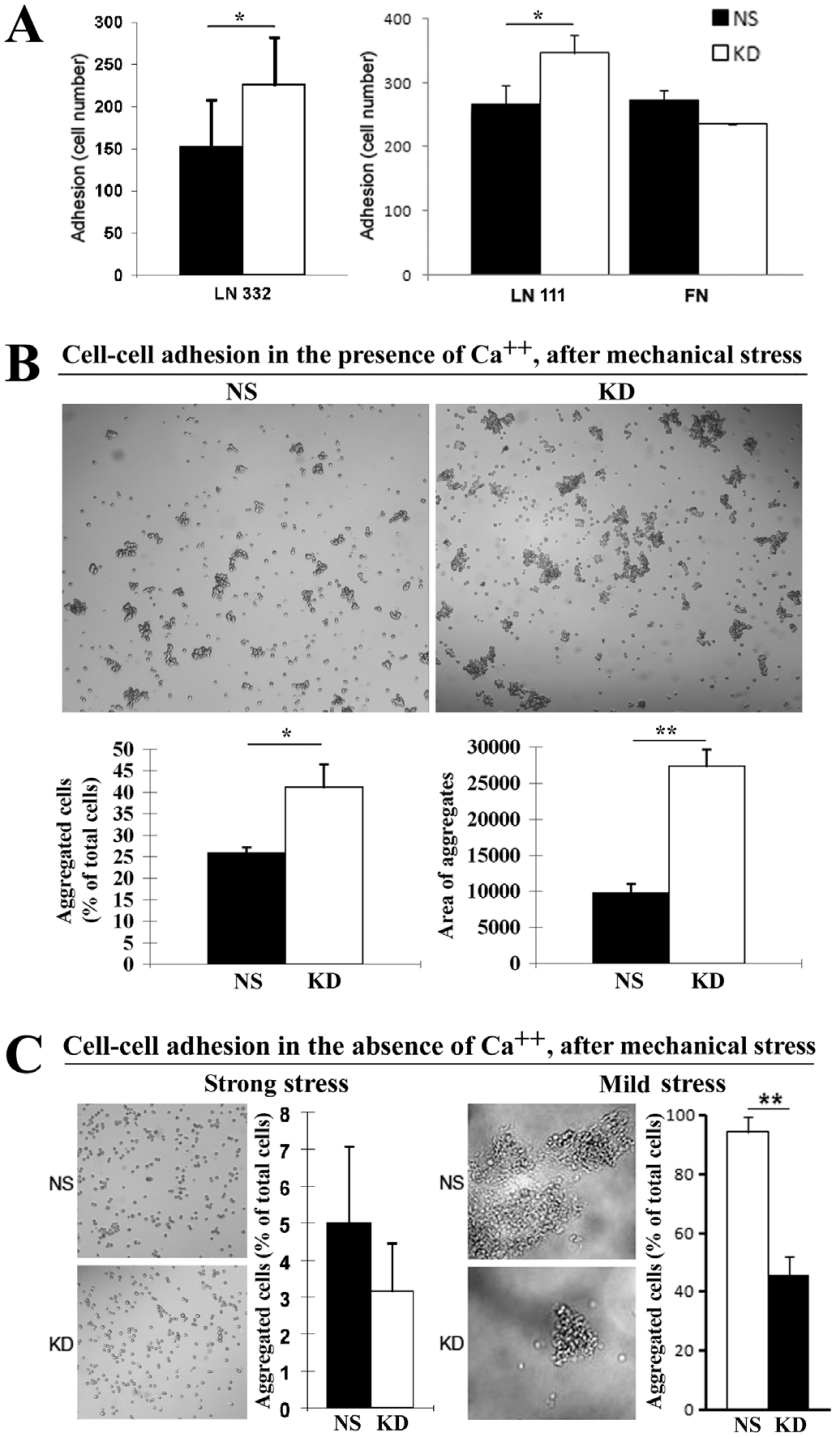
CO-029 regulated cell-matrix and cell-cell adhesions. (**A**) Cell-matrix adhesion. Adhesion onto laminin 111, laminin 332, and fibronectin of HT29-NS and -KD cells was assayed after incubating at 37^o^C in 5% CO_2_ for 1 h. The adhesion of the KD cells on laminin 111 and laminin 332 was significantly increased compared with NS cells (*P* = .042 on laminin 111 and  = .048 on laminin 332). (**B**) Total cell-cell adhesion. Cell aggregation was measured in the Ca^++^-containing media after a relatively strong mechanical force was applied. (**C**) Ca^++^-independent cell-cell adhesion. Cell aggregation was measured in the Ca^++^-free media after relatively strong or mild mechanical forces were applied, respectively. The aggregation of NS and KD cells after shear stress were quantified as described in Materials and Methods. *P* values are 0.03 for the aggregated cells in the presence of Ca^++^, 0.001 for the area of aggregates in the presence of Ca^++^, and 0.0004 for mild stress in the absence of Ca^++^. Images of cell aggregates after the shear-stress treatment were obtained under phase-contrast microscopy. All of the data are projected as mean±SEM (n = 4). *P<.05, **P<.01.

Tumor cell-matrix and cell-cell adhesions are considered to be the key events that regulate the metastatic cascade [32]–[34]. To evaluate the effect of CO-029 silencing on cell-matrix adhesion, we analyzed and compared the cell-matrix adhesiveness of KD and NS transductants on laminin and fibronectin. As shown in Figure 3A, HT29-NS and -KD cells displayed different adhesion abilities. The KD cells exhibited increased adhesiveness on extracellular matrix proteins laminin 111 and laminin 332, suggesting that CO-029 restrains cell adhesion on basement membrane, which is the matrix environment enriched in laminins. The cell adhesion on fibronectin, however, exhibited no differences between NS and KD cells (Figure 3A). We also examined cell adhesion on hyaluronan, a major proteoglycan of extracellular environment and the ligand of CD44, because of the decreased CD44 expression at the surface of HT29-KD cells. However, HT29 cells appeared not to adhere to hyaluronan, although an established protocol was followed for this assay [35].

To evaluate the effect of CO-029 silencing on cell-cell adhesion, we performed cell aggregation assay. First, we examined cell aggregation in the presence of Ca^++^ [28], which reflects the total cell-cell adhesiveness including both cadherin-dependent and -independent cell-cell adhesiveness. Silencing CO-029 resulted in increased ability to form the cell aggregates that are resistant to relatively strong mechanical stress, compared with NS cells (Figure 3B). Then, we examined cell-cell aggregation in the absence of Ca^++^, which reflects cadherin-independent cell-cell adhesiveness such as the one mediated by IgSF proteins. Ca^++^-independent cell-cell adhesion showed no difference after relatively strong mechanical stress was applied but was downregulated in KD cells after relatively mild mechanical force was applied (Figure 3B). In HT29 cells, Ca^++^-dependent cell-cell adhesion makes a major contribution to total cell-cell adhesion, based on the comparison between the magnitude of Ca^++^-dependent and -independent aggregates after strong mechanical stress treatment (Figure 3B). Together, CO-029 confines total and Ca^++^-dependent cell-cell adhesion.

### CO-029 Silencing Altered Cell Surface Expression Profile of Adhesion Molecules

**Figure 4:**
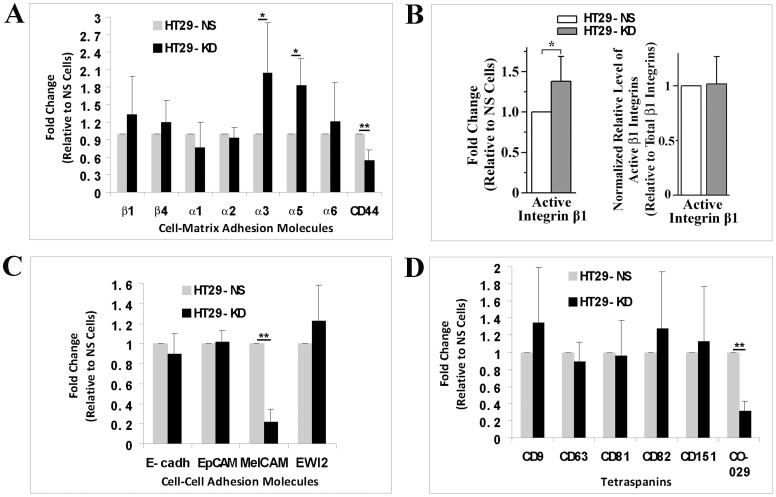
CO-029 silencing altered the surface expression of cell adhesion proteins and tetraspanins. The expression levels of cell-matrix adhesion proteins (**A**), active β1 integrins (**B**), cell-cell adhesion proteins (**C**), and tetraspanins (**D**) at the surface of HT29-NS and -KD transfectant cells were measured by flow cytometry. The relative levels of these proteins on KD cells to NS cells are presented as histograms (mean±SD, n = 4∼8). *P<.05, **P<.01. In (**B**), after normalized by the corresponding relative levels of total integrin β1, the levels of active integrin β1 relative to NS cells are presented in the histogram on the right.

The changes in the surface expression of tetraspanins, integrins, and cell adhesion proteins are involved in tumor progression and metastasis [17], [36]–[42]. To determine the mechanism by which CO-029 regulates cell-cell and -matrix adhesions, we measured and compared the expression levels of cell adhesion proteins on the surface of HT29-NS and -KD cells. For cell-matrix adhesion proteins, laminin receptor integrin α3 and fibronectin receptor integrin α5 were upregulated after silencing CO-029. Integrins β1, β4, α1, α2, and α6 remained unchanged, while hyaluronan receptor CD44 was markedly downregulated at the cell surface (Figure 4A). To determine whether CO-029 silencing affects integrin activation, we measured and compared the steady state levels of active β1 integrins in HT29-NS and -KD cells with flow cytometry by using β1 integrin mAb AG89. The β1 integrin mAb AG89 recognizes only the β1 integrins in active state [43]. Although the level of active β1 integrins was significantly increased at the cell surface of HT29-KD cells (Figure 4B left histogram), it remained unchanged after being calibrated with the level of total β1 integrins at the cell surface (Figure 4B right histogram). For cell-cell adhesion molecules, the level of E-cadherin proteins was not altered on the cell surface (Figure 4C). We also examined other cell-cell adhesion molecules related to CO-029 and/or tetraspanins such as EpCAM, MelCAM, and EWI2, which take part in Ca^++^-independent cell-cell adhesion and some of which belong to IgSF. Only MelCAM was markedly downregulated at the cell surface (Figure 4C). Tetraspanins CD9, CD63, CD81, CD82, and CD151 exhibited no significant alteration at the cell surface upon CO-029 silencing (Figure 4D).

### CO-029 and the Formation of Focal Adhesion, Stress Fiber, and Adherens Junction

**Figure 5:**
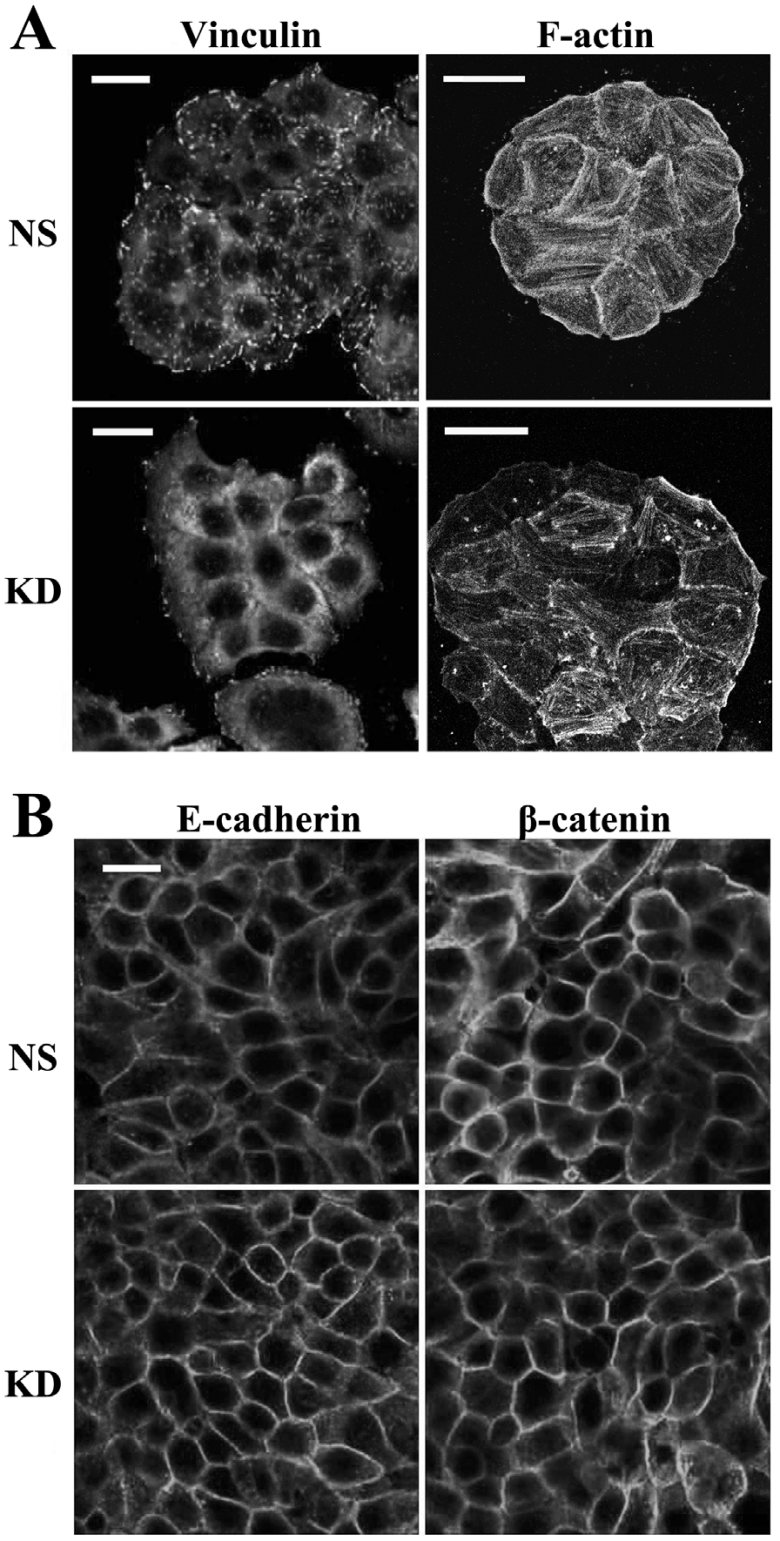
The effects of CO-029 silencing on the formations of focal adhesion, stress fiber, and adherens junction HT29 transfectants were plated on glass coverslips and cultured in complete media for 2 days (for vinculin and F-actin staining, **A**) or until confluence (for E-cadherin and β-catenin staining, **B**). The cells were fixed, permeabilized, and incubated with primary mAbs at 4°C overnight, followed by Alex Fluor 594-conjugated secondary Ab staining. F-actin was stained by Alex Fluor 594-conjugated phalloidin. The images were captured with confocal microscopy. Bar  = 20 µm.

To further evaluate the effect of CO-029 silencing on cell adhesion, we examined focal adhesion and adherens junction of the HT29 transfectant cells by staining i) vinculin, a marker of focal adhesion, and ii) E-cadherin and β-catenin, markers of adherens junction, respectively. We found that CO-029 silencing resulted in reduced formation of focal adhesion, as shown in Figure 5A. Meanwhile, the stress fiber formation near the basal surface of HT29-KD cells also became less robust compared with the one in HT29-NS cells (Figure 5A). In contrast, CO-029 silencing appears to have no disruptive effect on the formation of adherens junction, based on E-cadherin and β-catenin staining (Figure 5B). The cortical meshwork of actin along lateral surface or adherens junction exhibited no marked difference between NS and KD transfectant cells (data not shown).

### The Effect of CO-029 Silencing on the Formation of Tetraspanin-enriched Microdomain (TEM)

**Figure 6:**
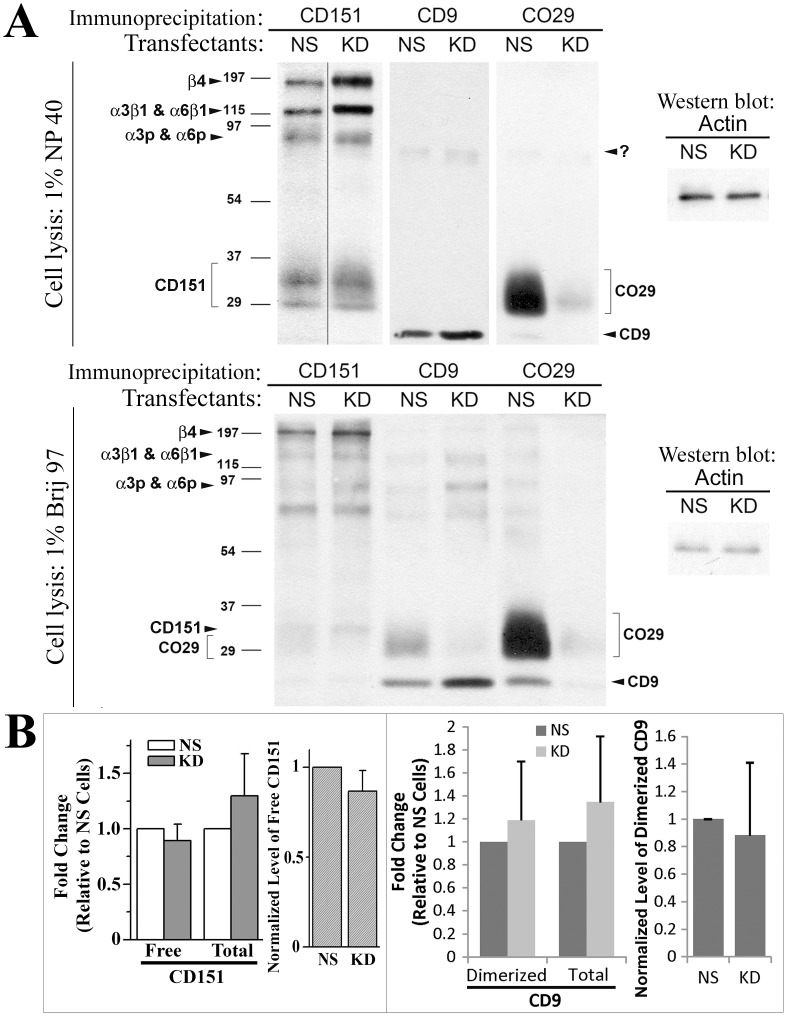
CO-029 silencing did not alter the formation of TEMs. (**A**) The surface biotinylated HT29-NS and -KD transfectant cells were lyzed with indicated detergent buffers. Tetraspanins CD9, CD151, and CO-029 were immunoprecipitated from the lysates, separated by SDS-PAGE, and detected by enhanced chemiluminescence. The levels of β-actin proteins in lysates were examined by Western blot and served as lysate input control. (**B**) The expression levels of integrin α3β1-unbound CD151, total CD151, homoclustered CD9, and total CD9 at the surface of HT29-NS and -KD transfectant cells were measured by mAb TS151r, 5C11, C9BB, and Mab7, respectively, using flow cytometry. The relative levels of CD151 and CD9 on KD cells to NS cells are presented as histograms (mean±SD, n = 3∼5).

Since CO-029 associates with tetraspanins such as CD9 and CD151 and laminin-binding integrins [11], we examined the effect of CO-029 silencing on the stability of TEM. The associations of CD151 with laminin-binding integrins α3β1, α6β1, and α6β4 remained stable under a stringent lysis condition, i.e., 1% Triton X100-mediated cell lysis, while the associations of CD151 with other tetraspanins remained stable under only a relatively mild lysis condition, i.e., 1% Brij 97-mediated cell lysis [3]. CO-029 silencing did not affect the immunoprecipitation profiles of CD151 under either lysis condition (Figure 6A). More laminin-binding integrins were co-precipitated with CD151 under the 1% NP40 lysis condition upon CO-029 silencing. Both CD151 and CD9 associated with CO-029 under the 1% Brij 97 lysis condition, as revealed by CD151 and CD9 immunoprecipitation profiles, while such associations were disrupted under 1% Triton X100 lysis condition, as expected. Under the 1% Brij 97 lysis condition, CO-029 immunoprecipitates also revealed CD9-CO-029 association but no CD151-CO-029 association because of less biotinylation of CD151 and co-migration of CD151 with CO-029 (Figure 6A).

Some of CD151 proteins at the cell surface bind integrin α3β1 in a direct protein-protein interaction manner [3]. We analyzed the level of integrin α3β1-unbound or “free” CD151 proteins, which are recognized by CD151 mAb TS151r [23]. At the cell surface, neither the level of free CD151 nor the total CD151-normalized level of free CD151 was changed upon CO-029 silencing (Figure 6B). Similarly, CD9 proteins at the cell surface are either homoclustered or associated with TEMs [21]. We found that neither the level of homoclustered CD9 nor the total CD9-normalized level of homoclustered CD9 was changed upon CO-029 silencing (Figure 6B). Together, these observations suggest that CO-029 is not required for the interaction of CD151 and CD9 with TEMs.

## Discussion

Tetraspanins regulate tumor progression and metastasis. But the mechanisms remain largely unknown. At the cellular level, tetraspanins modulate cell adhesion, migration, proliferation, and fusion. The adhesiveness and motility of tumor cells partially determine tumor metastatic potential. Hence, at the cellular level, tetraspanins probably regulate tumor metastasis by modulating the abilities of tumor cells to adhere and move. At the molecular level, tetraspanins associate with integrins, IgSF proteins, growth factors and their receptors, proteases, and intracellular signaling proteins to form TEM [44]–[47]. Hence, at the molecular level, tetraspanins regulate tumor progression and metastasis probably by altering the functions of the associated proteins [48]–[51].

The expression of tetraspanin CO-029 is typically linked to poor prognosis of digestive system cancers [12], [16], [18], [46], [52], [53], CO-029 is upregulated upon the progression of colorectal, liver, pancreatic, and esophageal cancers [11], [46], [54], and the increased expression of CO-029 promotes the liver or lung metastasis of these cancers [17], [52], [53], [55]. Tumor cell migration and invasion are indispensable for metastasis. The movement of tumor cells is involved in at least two phases in the metastasis cascade [52]. The first phase is that tumor cells migrate away from the primary tumor and invade the circulatory system; the second phase is that tumor cells migrate out of blood vessels and into target tissues. The reduced cell movement upon CO-029 silencing indicates that CO-029 is required for the efficient migration and invasion of colorectal cancer cells and suggests that CO-029 likely promotes both stages of tumor metastasis.

CO-029 appears to promote cell movement by altering cell-matrix and -cell adhesions. It is well established that cell-cell and -matrix adhesion directly determines cell motility. For example, the loss or reduction of E-cadherin expression and/or activity in tumor cells leads to the dispatch of tumor cells from primary tumor mass [56]. The increased total and Ca^++^-dependent cell-cell adhesion upon CO-029 silencing is consistent with the reduced motility of HT29-KD cells and also strongly suggests that CO-029 promotes cell movement by reducing cell-cell adhesion. The enhanced cell-cell adhesion, caused by CO-029 silencing, likely results from the increased activity of cadherins and/or other calcium-dependent cell-cell adhesion molecules. E-cadherin, however, appears to be unaltered because the cell surface level of E-cadherin and the recruitment of E-cadherin to adherens junction remain unchanged. In collective cell migration such as wound healing, cell-cell adhesion directly regulates cell migration; and increased calcium-dependent cell-cell adhesiveness upon CO-029 silencing is likely to be responsible for or at least contribute to the decreased collective migration or healing process of HT29-KD cells. While in solitary cell migration such as transwell cell migration, the increased surface presence of integrins α3β1 and α5β1 and upregulated activity of integrins α6β1 and/or α6β4 probably cause decreased migration onto extracellular matrices of HT29-KD cells.

For cell-matrix adhesion, the selectively increased adhesion on laminins but not on fibronectin upon CO-029 silencing suggests that CO-029 likely inhibits cell adhesion on laminins. Because laminins are the constituents of basement membranes that typically keep epithelial and endothelial cells in stationary, the role of CO-029 in inhibiting cell adhesion on laminins is consistent with its role in facilitating cell movement. The increased adhesion on laminin 111, which is ubiquitously expressed in epithelia, implies the activation of its receptors of integrins α6β1 and/or α6β4 because the surface levels of these integrins were not altered in HT29-KD cells, while increased adhesion on laminin 332, which is found mainly in skin, intestine, respiratory, and urinary epithelia, likely reflects the enhanced surface level of its receptor integrin α3β1, which is the major component of TEMs, and probably also the enhanced activity of its receptor integrin α6β4. Although the level of fibronectin-binding integrin α5β1 at the cell surface is upregulated upon CO-029 silencing, cell adhesion on fibronectin was not altered in HT29-KD cells, which is presumably due to a reduced functional activity of integrin α5 β 1 or lower levels and/or activities of other fibronectin-binding integrins like αV integrins. The altered levels of cell adhesion proteins at the cell surface upon CO-029 silencing could result from the altered endocytosis and/or recycling of these transmembrane proteins. In other words, CO-029 probably regulates the trafficking of these proteins through TEMs.

Since the levels of α6 integrins remain unaltered upon CO-029 silencing, the increased cell adhesion onto laminins observed in our study could result from the upregulated activity of integrin α6β4. Because HT-29 colon cancer cells form hemidesmosomes [57], [58] and integrin α6β4 is the only integrin in hemidesmosomes, the upregulated activity of integrin α6β4 may lead to the formation of stronger or more hemidesmosomes. In addition to the cell-matrix adhesive structures that can be visualized microscopically such as focal adhesions and hemidesmosomes, cell-matrix adhesion is also mediated by the direct engagement to matrices of the cell adhesion molecules that do not form microscopic adhesive structure. This type of cell-matrix adhesion mechanism is diffusely and evenly distributed at the interface of the basal plasma membrane of a cell and the underlying matrices, while focal adhesions and hemidesmosomes are spotted at this interface. Hence, the increased adhesiveness onto laminins could also reflect the upregulation in level or activity of the laminin-binding integrins α3β1, α6β1, and α6β4 that do not form microscopic adhesive structures.

Reduced cell adhesion at the early stage of metastasis helps release tumor cells from the primary tumor. Cell-cell adhesion needs to be disrupted so that invasive tumor cells can dissociate from the primary tumor and infiltrate interstitial tissue. Thus, cell-cell adhesion is an important factor in tumor cell invasiveness and metastasis [59]–[61]. In addition, tumor cells need to reduce the adhesiveness on laminins to effectively breach or pass through various epithelium and endothelium basement membranes that tumor cells encounter during metastasis. Therefore, we extrapolate that CO-029 likely facilitates 1) the dispatch of tumor cells by reducing cell-cell adhesion at the early stage of metastasis, and 2) the infiltration, intravasation, and extravasation of tumor cells by confining the adhesion on laminins in the basement membranes of epithelium and endothelium. Moreover, CO-029 perturbs other molecules, such as CD44 and MelCAM, important for metastasis. Besides directly engaging cell-matrix and -cell adhesions, CD44 facilitates cancer progression and serves as a marker for cancer-initiating cells [62]–[66]. CD44 could be one of the key molecules through which CO-029 promotes cell motility and cancer metastasis, especially given that CO-029 and CD44 form a complex in colorectal cancer cells and correlate with the progression of this cancer [9].

In summary, the cell migration capability is markedly diminished upon the silencing of CO-029. Because CO-029 regulates cell-cell and cell-matrix adhesions, the reduced motility of HT29-KD cells likely results from the deregulated cell adhesions. The altered cell-matrix adhesion is caused by the altered surface expression or activity of integrins and CD44, while the altered cell-cell adhesion is caused mainly by the malfunction of calcium-dependent cell-cell adhesion molecules (Figure S2). Hence, CO-029 likely promotes the progression and metastasis of colorectal cancer by enhancing tumor cell movement and deregulating cell adhesions.

## Supporting Information

Figure S1 **CO-029 silencing impaired colorectal cancer cell migration.** (A) Wound healing assay. Compared with HT29-NS transfectant cells, wound closure was significantly impaired in HT29-KD2 transfectant cells at 72 h after wounds were generated in confluent cell monolayers. (B) Transwell migration assay. The KD2 transfectant cells exhibited reduced motility in Transwell migration experiments. n = 3. *P<.05.
(TIF)

Figure S2 **Schematic representation of the effect of CO-029 silencing on cell adhesion proteins.**
(TIF)

Materials & Methods S1 **Supplemental Materials and Methods.**
(DOC)
