# Supplementary figures and images for: Tetraspanin CO-029 Inhibits Colorectal Cancer Cell Movement by Deregulating Cell-Matrix and Cell-Cell Adhesions
Source: PLoS One. 2012 Jun 5;7(6):e38464. doi: 10.1371/journal.pone.0038464 (PMC3367972; doi:10.1371/journal.pone.0038464)

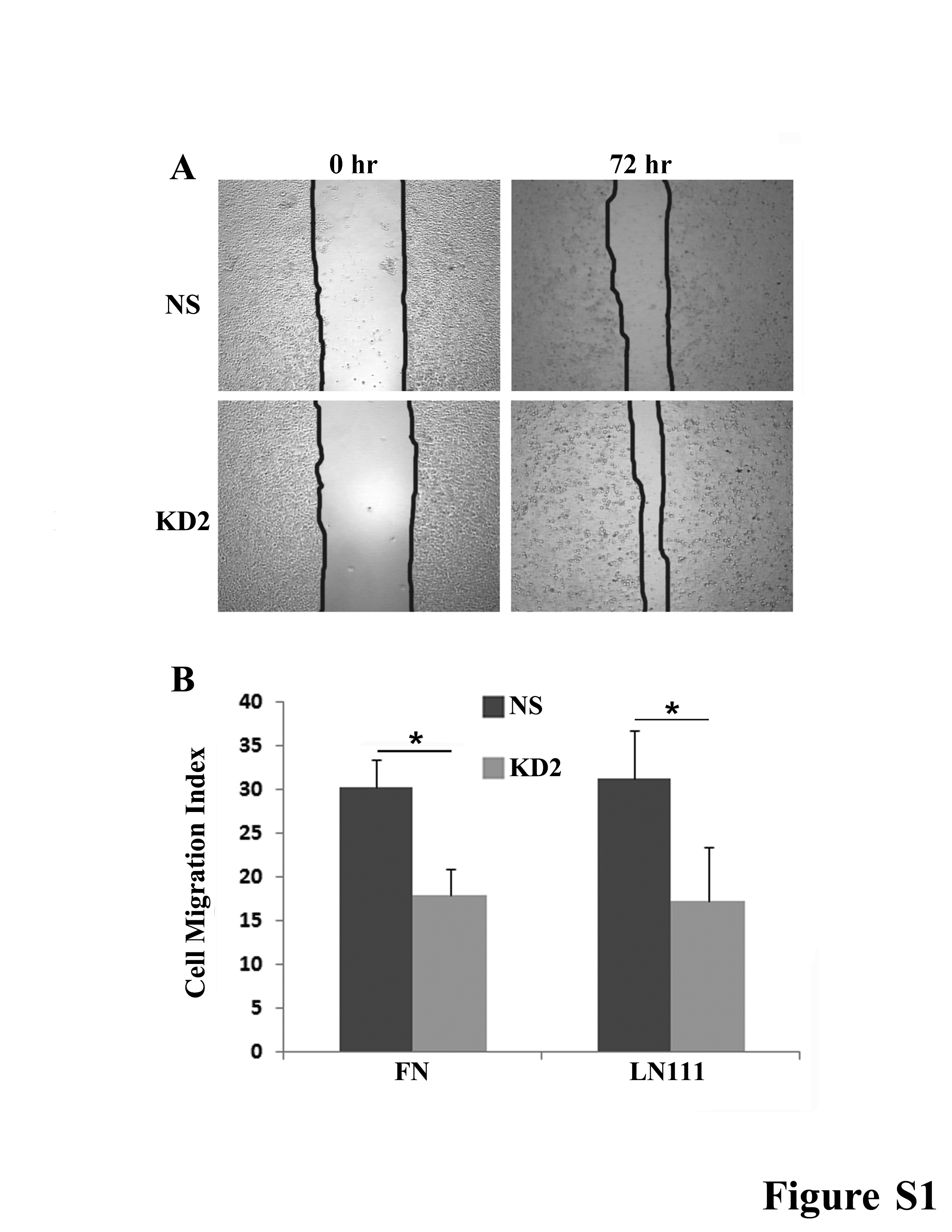

Supplement: Figure S1 — CO-029 silencing impaired colorectal cancer cell migration. (A) Wound healing assay. Compared with HT29-NS transfectant cells, wound closure was significantly impaired in HT29-KD2 transfectant cells at 72 h after wounds were generated in confluent cell monolayers. (B) Transwell migration assay. The KD2 transfectant cells exhibited reduced motility in Transwell migration experiments. n = 3. *P<.05. (TIF) [file pone.0038464.s001.tif]

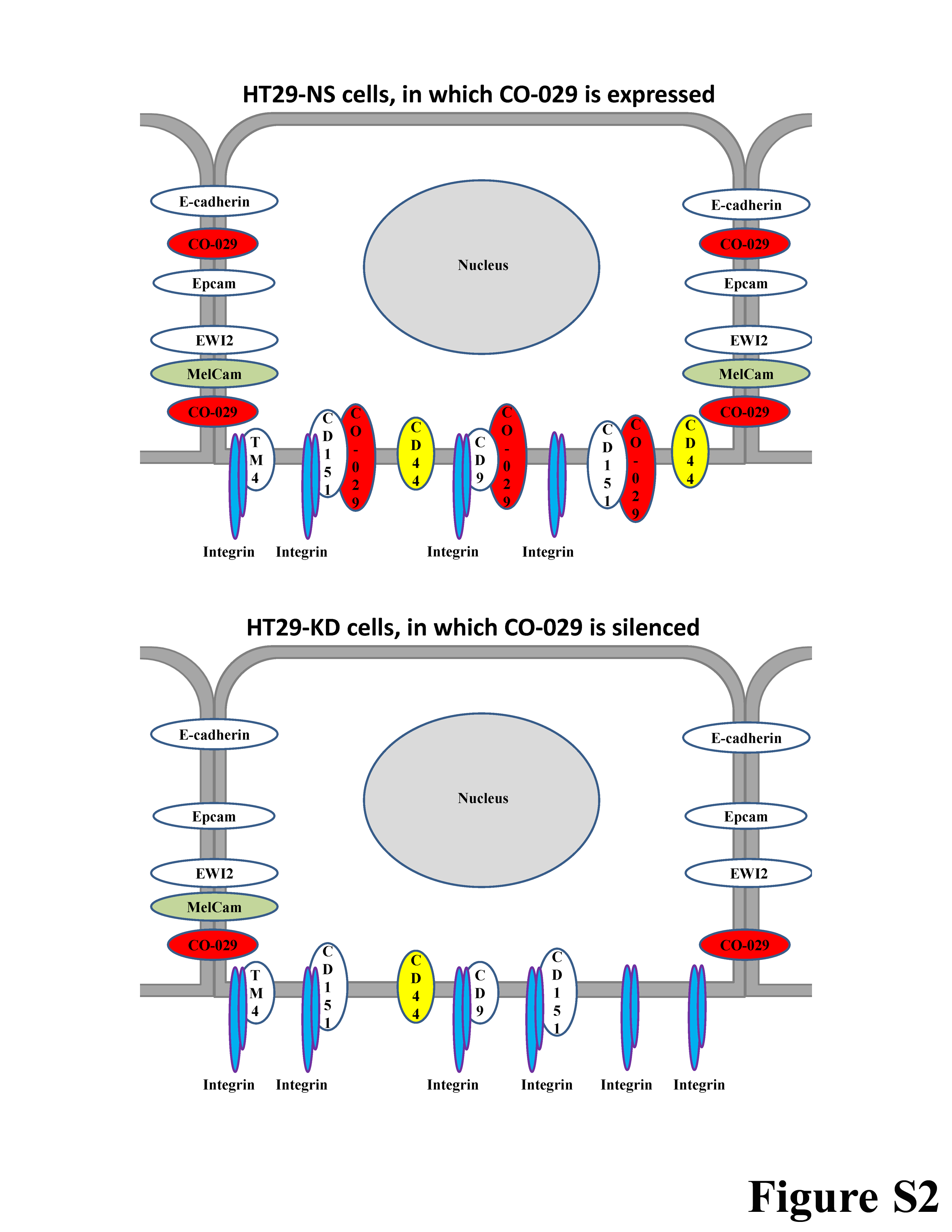

Supplement: Figure S2 — Schematic representation of the effect of CO-029 silencing on cell adhesion proteins. (TIF) [file pone.0038464.s002.tif]
