# Supplementary material for: Tetraspanin CO-029 Inhibits Colorectal Cancer Cell Movement by Deregulating Cell-Matrix and Cell-Cell Adhesions
Source: PLoS One. 2012 Jun 5;7(6):e38464. doi: 10.1371/journal.pone.0038464 (PMC3367972; doi:10.1371/journal.pone.0038464)
Supplement: Materials & Methods S1 — Supplemental Materials and Methods. (DOC) [file pone.0038464.s003.doc]

**Supplemental Materials and Methods**

RNA Interference

The CO-029 small hairpin RNA (shRNA) (TGCTGTTGACAGTGAGCGAAGAGACCATGCCAAAGCTATATAGTGAAGCCACAGATGTATATAGCTTTGGCATGGTCTCTGTGCCTACTGCCTCGGA), which targets the AGAGACCATGCCAAAGCTAT sequence of human CO-029 mRNA, was used to generate CO-029 KD2 transfectant in HT29 cells, as described in “Materials and Methods”.
